# Supplementary material for: Real-world size of objects serves as an axis of object space
Source: Commun Biol. 2022 Jul 27;5:749. doi: 10.1038/s42003-022-03711-3 (PMC9329427; doi:10.1038/s42003-022-03711-3)
Supplement: Supplementary file 2 — Supplementary Materials [file 42003_2022_3711_MOESM2_ESM.pdf]

Supplementary Materials for

Real-world size of objects serves as an axis of object space

Taicheng Huang, Yiying Song*, Jia Liu*

*Corresponding author. Email: songyiying@bnu.edu.cn, liujiathu@tsinghua.edu.cn

Figure S1.

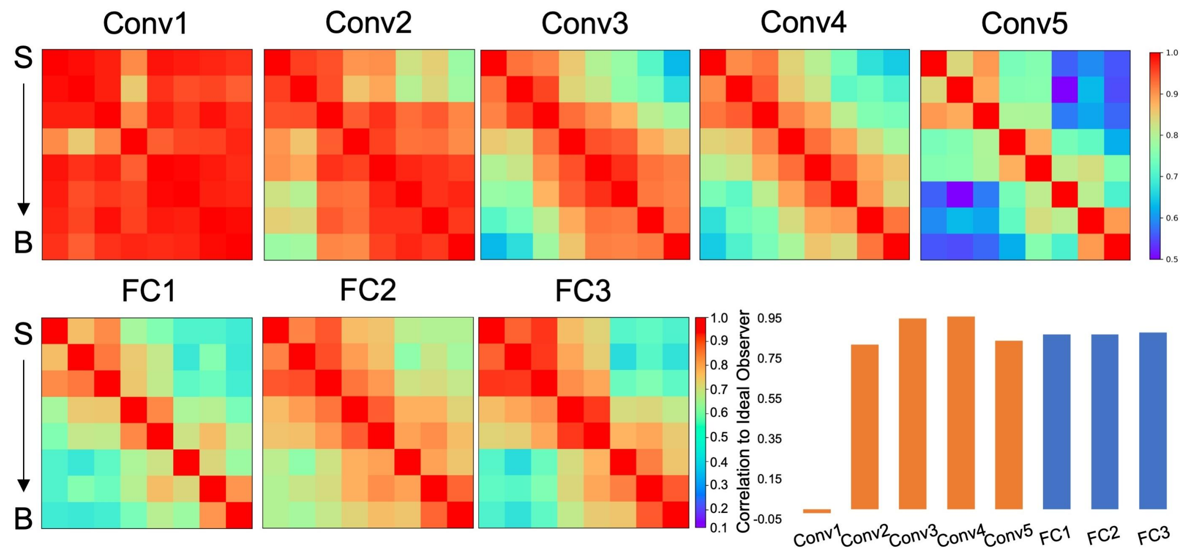

Fig S1. Representational similarity matrix (RSM) of size ranks in different layers of AlexNet. The RSM showed high correspondence to the ideal observer from Conv2 layer, and achieved the highest value in Conv4 layer. S: small objects; B: big objects.

Figure S2.

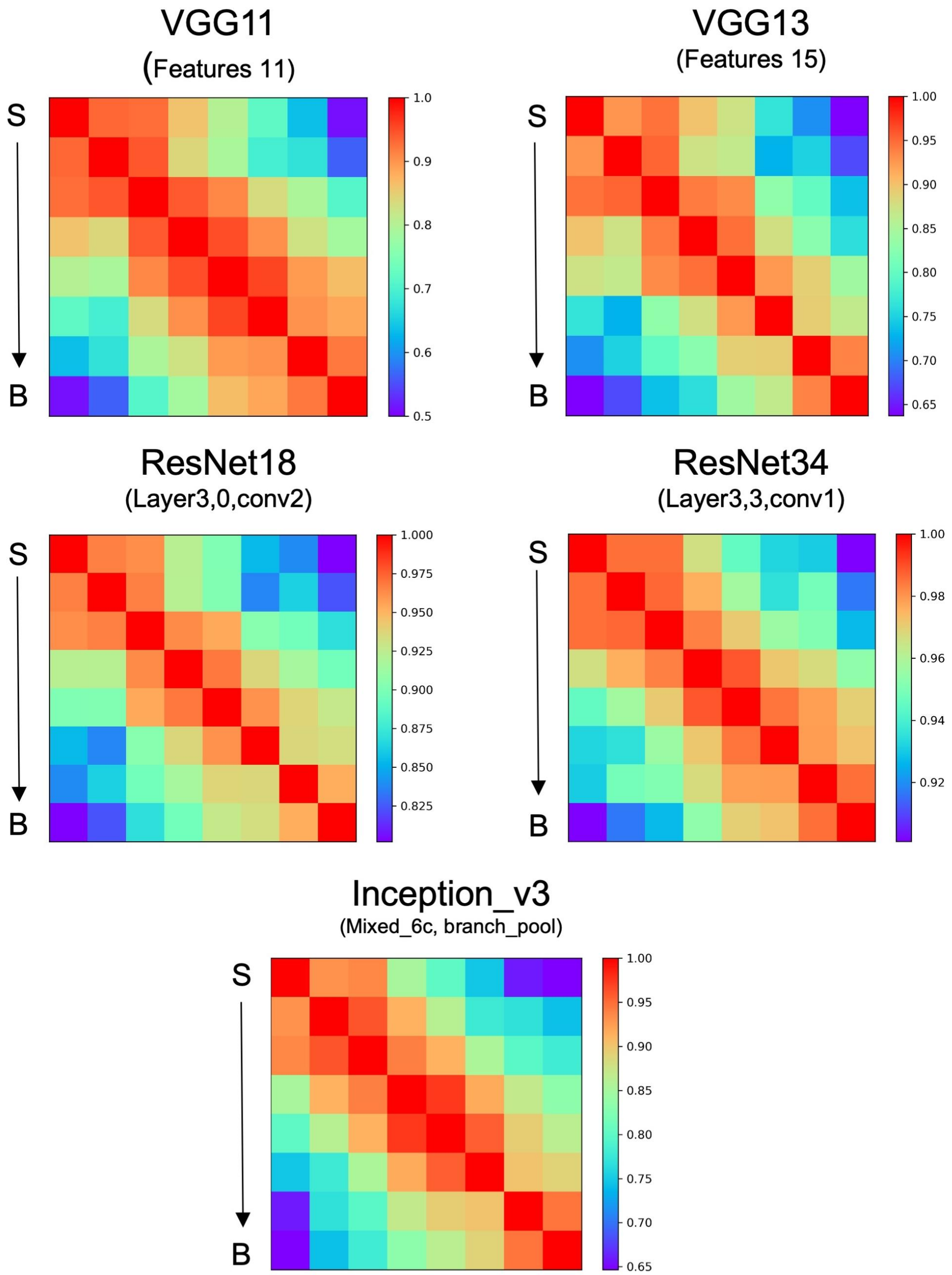

Fig S2. The representation of objects' real-world size was ubiquitously observed in DCNNs with different architectures. Five representative implementations, including two from VGG family, two from ResNet family, and one from Inception family were examined. Their correlations to the ideal observer were 0.95 (VGG11), 0.96 (VGG13), 0.96 (ResNet18), 0.96 (ResNet34) and 0.97 (Inception_v3), respectively. S: small objects; B: big objects.

Figure S3.

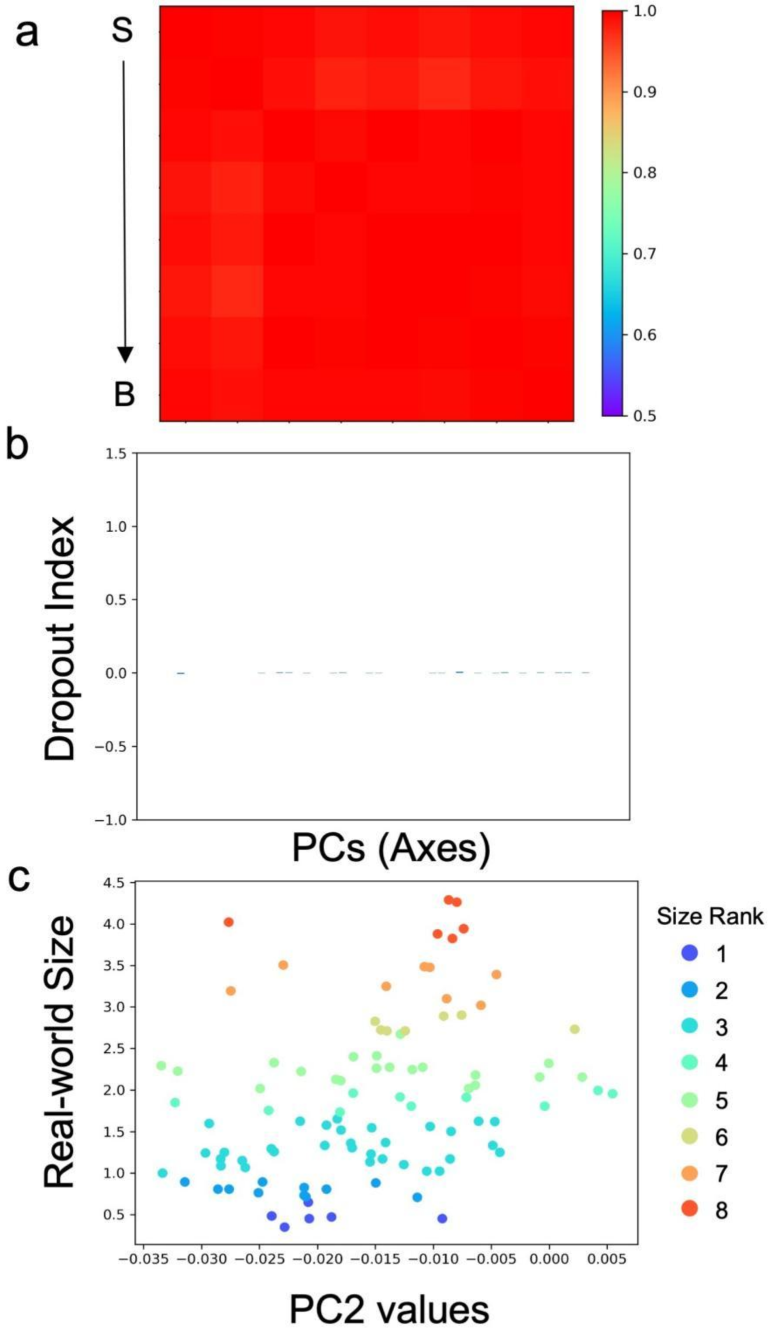

Fig S3. Untrained AlexNet showed no representation for objects' real-world size. (a) The RSM of size ranks in untrained AlexNet was unable to distinguish object sizes. (b) No principal axis for objects' real-world size was found in untrained AlexNet. (c) No logarithm correspondence between PC2 values and objects' real-world size. S: small objects; B: big objects.

Figure S4

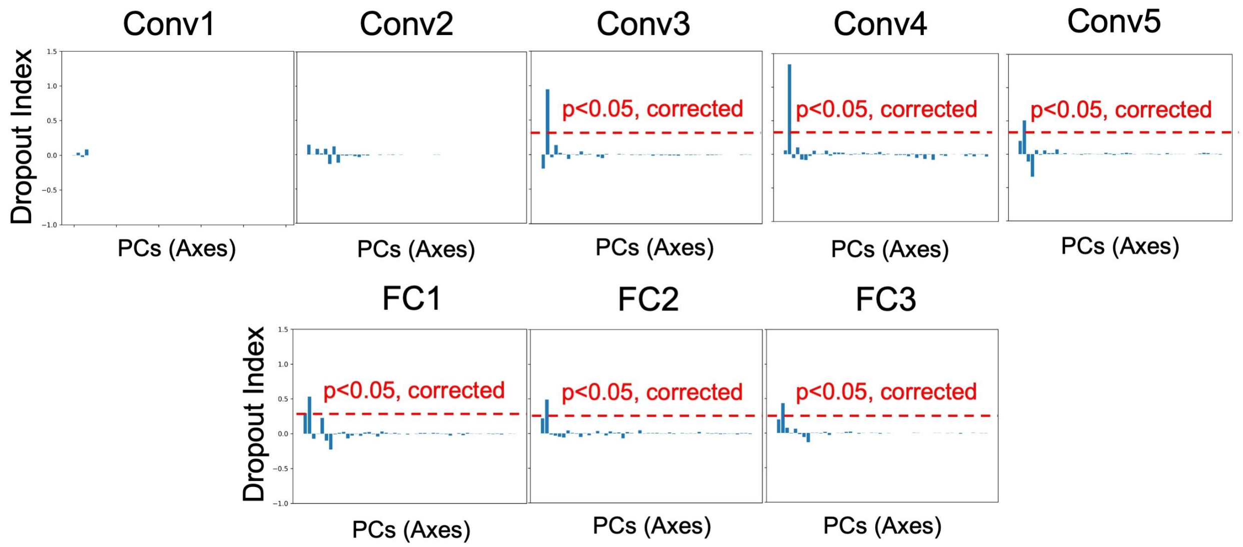

Fig S4. Layer-wise analyses on the selectivity of PC2 in encoding objects' real-world size. The encoding of objects' size started from Conv3 layer to FC3 layer. Note that this feature was selectively encoded in PC2 in all of these layers.

Figure S5.

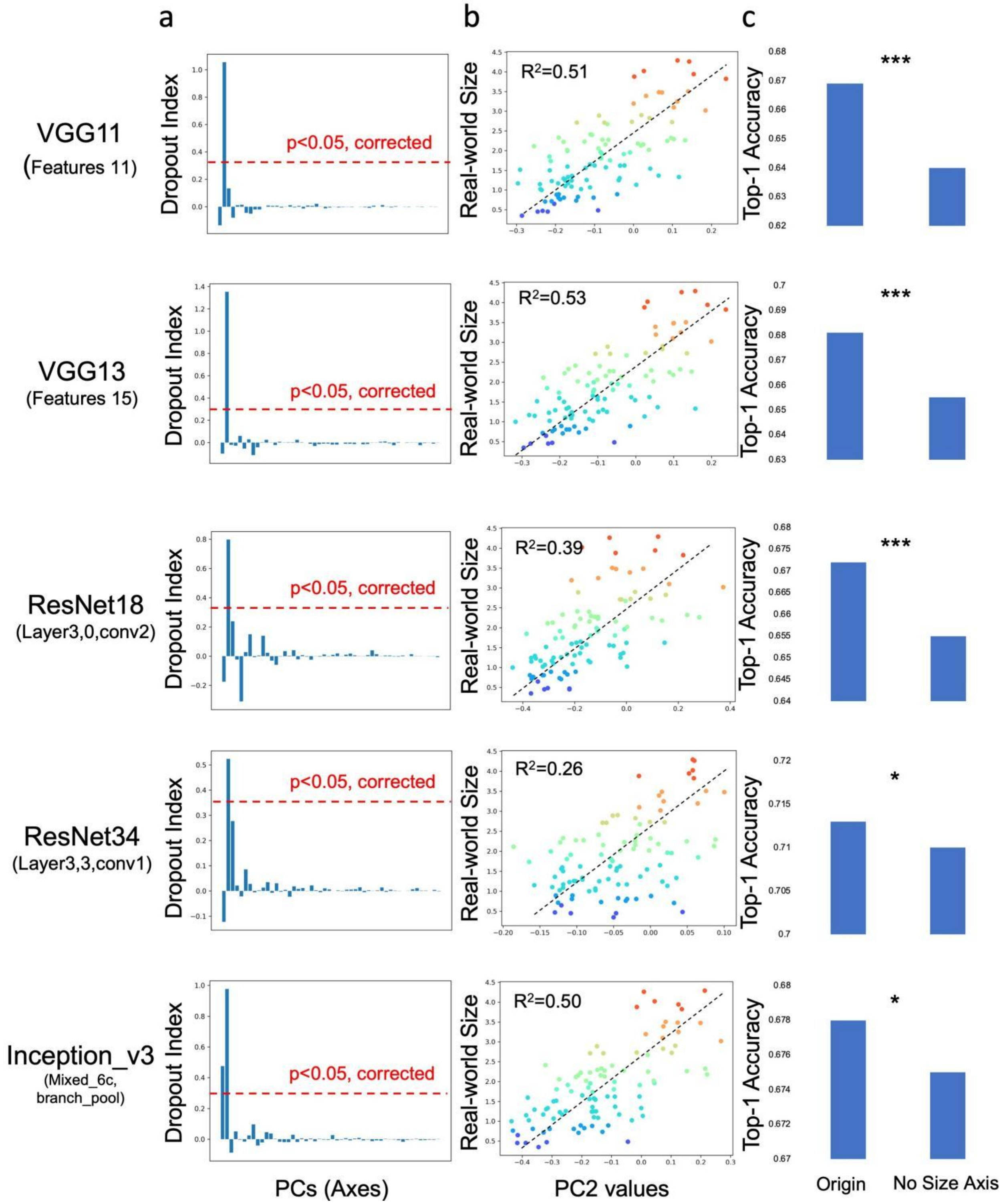

Fig S5. The size axis was observed in DCNNs with different architectures. (a) The size axis was present in five different DCNNs, including two VGG networks, two ResNet networks, and one Inception network. (b) Logarithm correspondence between PC2 and objects' size of DCNNs. (c)

The top-1 accuracy decreased significantly when the variance of the size axis was removed from DCNNs. *, $p<0.05$; ***, $p<0.001$.

Figure S6.

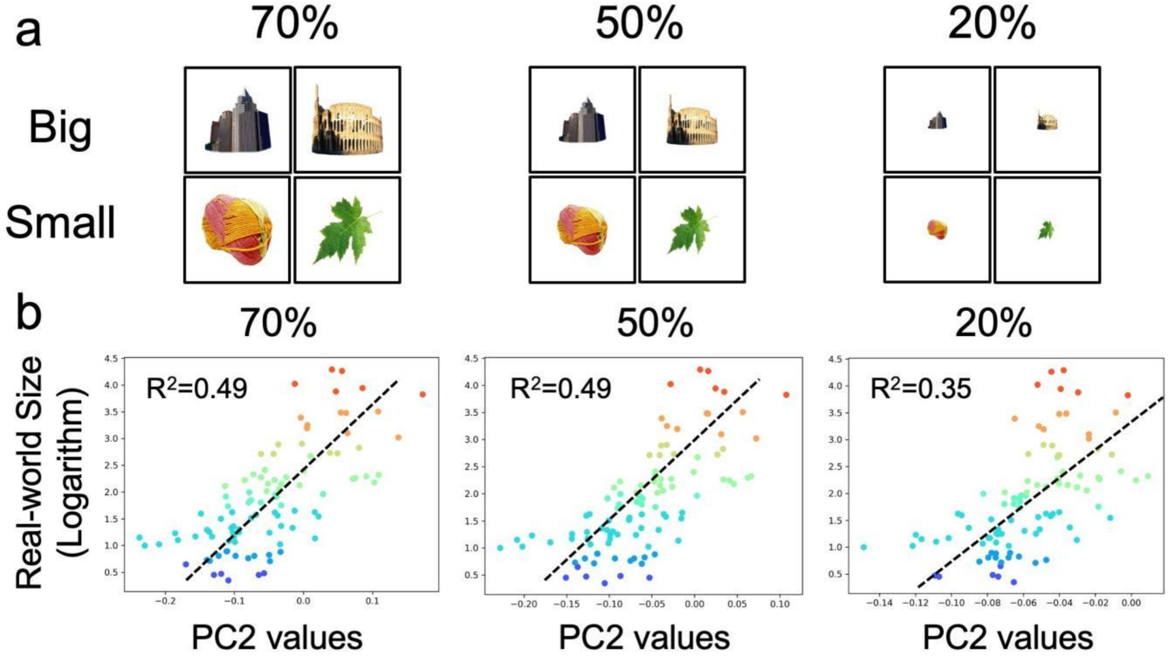

Fig S6. The size axis was tolerant to the absolute retinal size of objects. (a) Illustration of stimuli used for testing whether absolute retinal size was used to infer object's size. Objects were shrunk to 70%, 50%, 20% of the original images. (b) Common logarithm correspondence between PC2 and real-world size was relatively stable.

Table S1.

Size Rank	Size Range	Number of Objects	Representative objects
1	1-5	6	Button, Dice, Thumbtack
2	5-10	11	Key, Garlic, Teabag
3	10-50	32	Shoe, Mouse, Helmet
4	50-100	10	Backpack, Screen, Cooler
5	100-500	20	Grill, Refrigerator, Sofa
6	500-1,000	7	Car, Statue, Fountain
7	1,000-5,000	8	Monument, Lighthouse, Freight train
8	5,000+	6	Airplane, Colosseum, Arch of Triumph

Table S1. Size ranks of objects. One hundred background-free objects were grouped into eight size ranks to control confounding factors which were unrelated to real-world size. All size ranks contain no less than six objects. The unit of size range is centimeter.
