# Supplementary material for: Real-world size of objects serves as an axis of object space
Source: Commun Biol. 2022 Jul 27;5:749. doi: 10.1038/s42003-022-03711-3 (PMC9329427; doi:10.1038/s42003-022-03711-3)
Supplement: Supplementary file 3 — Description of Additional Supplementary Files [file 42003_2022_3711_MOESM3_ESM.pdf]

Description of Additional Supplementary Files

File name: Supplementary Data 1
Description: The source data of representational similarity matrices for the graphs (i.e., Figure 1, 3, 4, 5, 7) in the paper

File name: Supplementary Data 2
Description: The source data of PC2 values and the real-world size of objects for the graphs (i.e., Figure 2, 3, 4, 5, 7) in the paper.

File name: Supplementary Data 3
Description: The source data of the dropout index for the graphs (i.e., Figure 2, 3, 4, 7) in the paper.
