# Supplementary material for: Real-world size of objects serves as an axis of object space
Source: Commun Biol. 2022 Jul 27;5:749. doi: 10.1038/s42003-022-03711-3 (PMC9329427; doi:10.1038/s42003-022-03711-3)
Supplement: Supplementary file 7 — Reporting Summary [file 42003_2022_3711_MOESM7_ESM.pdf]

nature research

Corresponding author(s): Jia Liu

Last updated by author(s): 6/26/2022

Reporting Summary

Nature Research wishes to improve the reproducibility of the work that we publish. This form provides structure for consistency and transparency in reporting. For further information on Nature Research policies, see our Editorial Policies and the Editorial Policy Checklist.

Statistics

For all statistical analyses, confirm that the following items are present in the figure legend, table legend, main text, or Methods section.

n/a	Confirmed	
☐	☒	The exact sample size (n) for each experimental group/condition, given as a discrete number and unit of measurement
☐	☒	A statement on whether measurements were taken from distinct samples or whether the same sample was measured repeatedly
☐	☒	The statistical test(s) used AND whether they are one- or two-sided<br>*Only common tests should be described solely by name; describe more complex techniques in the Methods section.*
☐	☒	A description of all covariates tested
☐	☒	A description of any assumptions or corrections, such as tests of normality and adjustment for multiple comparisons
☐	☒	A full description of the statistical parameters including central tendency (e.g. means) or other basic estimates (e.g. regression coefficient) AND variation (e.g. standard deviation) or associated estimates of uncertainty (e.g. confidence intervals)
☐	☒	For null hypothesis testing, the test statistic (e.g. F, t, r) with confidence intervals, effect sizes, degrees of freedom and P value noted<br>*Give P values as exact values whenever suitable.*
☒	☐	For Bayesian analysis, information on the choice of priors and Markov chain Monte Carlo settings
☒	☐	For hierarchical and complex designs, identification of the appropriate level for tests and full reporting of outcomes
☐	☒	Estimates of effect sizes (e.g. Cohen's d, Pearson's r), indicating how they were calculated

Our web collection on statistics for biologists contains articles on many of the points above.

Software and code

Policy information about availability of computer code

Data collection	The datasets presented in this study can be found in Online repositories, the name of each repository and the download location can be found in the article.
Data analysis	Data were analyzed using custom Python code. All code and data of our study necessary for reproducing our results are available on Github: https://github.com/helloTC/RealWorldSizeAxis. The single-object version of the ImageNet ILSVRC2012 dataset is available on ScienceDB: https://www.scidb.cn/en/doi/10.57760/sciencedb.01674. Other datasets presented in this study can be found in Online repositories, and the name of each repository and the download location can be found in the article.

For manuscripts utilizing custom algorithms or software that are central to the research but not yet described in published literature, software must be made available to editors and reviewers. We strongly encourage code deposition in a community repository (e.g. GitHub). See the Nature Research guidelines for submitting code & software for further information.

Data

Policy information about availability of data

All manuscripts must include a data availability statement. This statement should provide the following information, where applicable:

- Accession codes, unique identifiers, or web links for publicly available datasets
- A list of figures that have associated raw data
- A description of any restrictions on data availability

All code and data of our study necessary for reproducing our results are available on Github: https://github.com/helloTC/RealWorldSizeAxis. The single-object version of the ImageNet ILSVRC2012 dataset is available on ScienceDB: https://www.scidb.cn/en/doi/10.57760/sciencedb.01674. Other datasets presented in this study can be found in Online repositories, and the name of each repository and the download location can be found in the article.

Field-specific reporting

Please select the one below that is the best fit for your research. If you are not sure, read the appropriate sections before making your selection.

☒ Life sciences ☐ Behavioural & social sciences ☐ Ecological, evolutionary & environmental sciences

For a reference copy of the document with all sections, see nature.com/documents/nr-reporting-summary-flat.pdf

Life sciences study design

All studies must disclose on these points even when the disclosure is negative.

Sample size	The sample size for the fMRI experiment is 10 because all participants showed a consistent pattern (see Fig 5.). The sample size for behavioral experiment is 2 because the task is extremely easy and all participants showed a consistent result (r > 0.90).
Data exclusions	No data was excluded.
Replication	The findings from AlexNet were replicated with other DCNNs, such as VGG11, VGG13, ResNet18, ResNet34 and Inception_V3.
Randomization	All samples used for DCNN were directly downloaded from the Internet and all datasets are widely used. the participants for the fMRI experiment were randomly recruited from Beijing Normal University, Beijing, China.
Blinding	Yes.

Reporting for specific materials, systems and methods

We require information from authors about some types of materials, experimental systems and methods used in many studies. Here, indicate whether each material, system or method listed is relevant to your study. If you are not sure if a list item applies to your research, read the appropriate section before selecting a response.

Materials & experimental systems

n/a	Involved in the study	
☒	☐	Antibodies
☒	☐	Eukaryotic cell lines
☒	☐	Palaeontology and archaeology
☒	☐	Animals and other organisms
☐	☒	Human research participants
☒	☐	Clinical data
☒	☐	Dual use research of concern

Methods

n/a	Involved in the study	
☒	☐	ChIP-seq
☒	☐	Flow cytometry
☐	☒	MRI-based neuroimaging

Human research participants

Policy information about studies involving human research participants

Population characteristics	Ten participants (5 males and 5 females, age range: 18-27 years) from Beijing Normal University, Beijing, China, participated in this study to examine the effects of shape or texture on the real-world size representation in the human brain. All participants had a normal or corrected-to-normal vision. Informed consent was obtained according to procedures approved by the Institutional Review Board of Beijing Normal University.
Recruitment	Participants were recruited through an Internet advertising system.
Ethics oversight	The Institutional Review Board of Beijing Normal University.

Note that full information on the approval of the study protocol must also be provided in the manuscript.

Magnetic resonance imaging

Experimental design

Design type	Blocked design
Design specifications	Each run consisted of six conditions (i.e., big origin, small origin, big silhouette, small silhouette, big texture, and small texture). Each run lasted 320s, which included four blocks. Each block lasted 60s, consisting of three conditions with all 40 images per condition. Each image was presented for 200ms, followed by a 300ms fixation.

Behavioral performance measures	A passive-view task was employed, and therefore no behavioral performance in the fMRI experiment was recorded.

Acquisition

Imaging type(s)	Functional and structural images
Field strength	3T
Sequence & imaging parameters	The anatomical images were acquired with a magnetization-prepared rapid gradient-echo (MPRAGE) sequence. Parameters for the T1 image are: TR/TE=2,530/2.27ms, flip angle=7°, voxel resolution=1×1×1mm. Blood oxygenation level-dependent (BOLD) contrast was obtained with a gradient echo-planar T2* sequence. Parameters for the T2* image are: TR/TE=2,000/34.0ms, flip angle=90°, voxel resolution=2×2×2mm, FoV=200×200mm.
Area of acquisition	Whole brain

Diffusion MRI ☐ Used ☒ Not used

Preprocessing

Preprocessing software	fMRIPrep (version 20.2.0) and ciftify.
Normalization	A non-linear normalization was used to project images into a 32k_fs_LR space.
Normalization template	MNI152
Noise and artifact removal	ICA-AROMA
Volume censoring	fMRIPrep

Statistical modeling & inference

Model type and settings	Univariate and Multivariate analyses.
Effect(s) tested	The difference between objects' shape and texture in inferring objects' real-world size.

Specify type of analysis: ☐ Whole brain ☐ ROI-based ☒ Both

Anatomical location(s)	We used the half runs to define the size-sensitive ROIs and the examined the hypothesis using the other half.
Statistic type for inference (See Eklund et al. 2016)	voxel-wise
Correction	FWE

Models & analysis

n/a	Involved in the study
☒	☐ Functional and/or effective connectivity
☒	☐ Graph analysis
☒	☐ Multivariate modeling or predictive analysis
